# Prophylactic treatment of FSGS recurrence in patients who relapsed on a previous kidney graft

**DOI:** 10.1093/ndt/gfae108

**Published:** 2024-05-24

**Authors:** Charlotte Uro-Coste, Céline Lambert, Vincent Audard, Lionel Couzi, Sophie Caillard, Matthias Büchler, Arnaud Del Bello, Paolo Malvezzi, Vincent Pernin, Charlotte Colosio, Laurent Mesnard, Dominique Bertrand, Frank Martinez, Didier Ducloux, Coralie Poulain, Antoine Thierry, Clément Danthu, Clarisse Greze, Camille Lanaret, Valérie Moal, Alexandre Hertig, Jacques Dantal, Christophe Legendre, Valérie Chatelet, Antoine Sicard, Clément Gosset, Nicolas Maillard, Agnès Duveau, Clémence Petit, Nassim Kamar, Anne-Elisabeth Heng, Dany Anglicheau, Cyril Garrouste

**Affiliations:** Service de Néphrologie, CHU Clermont-Ferrand, Clermont-Ferrand, France; Unité de Biostatistiques, DRCI, CHU Clermont-Ferrand, Clermont-Ferrand, France; Assistance Publique des Hôpitaux de Paris, Service de Néphrologie et Transplantation Centre de Référence Maladie Rare « Syndrome Néphrotique Idiopathique », Hôpitaux Universitaires Henri-Mondor, Univ Paris Est Créteil, INSERM, IMRB, Créteil, France; Service de Néphrologie, Transplantation, Dialyse et Aphérèses, CHU de Bordeaux, Bordeaux, France; Service de Néphrologie, University Hospital, Strasbourg, France; Service de Néphrologie et Immunologie Clinique, CHRU de Tours, Tours, France; Département de Néphrologie et Transplantation d'Organes, CHU Toulouse, INSERM U1043, IFR–BMT, Université Paul Sabatier, Toulouse, France; Service de Néphrologie, Hémodialyse, Aphérèses et Transplantation Rénale, CHU Grenoble-Alpes, Grenoble, France; Service de Néphrologie, Dialyse et Transplantation, Hôpital Lapeyronie, CHU Montpellier, Montpellier, France; Service de Néphrologie et Transplantation, CHU Reims, Reims, France; Assistance Publique – Hôpitaux de Paris, Soins Intensifs Néphrologiques et Rein Aigu, APHP Sorbonne Université, Hôpital Tenon, Paris, France; Service de Néphrologie, CHRU, Rouen, France; Assistance Publique des Hôpitaux de Paris, Service de Néphrologie et Transplantation, Hôpital Universitaire Necker-Enfants Malades, Université de Paris, Paris, France; Service de Néphrologie, Dialyse et Transplantation, CHU Besançon, Besançon, France; Service de Néphrologie-Médecine Interne-Dialyse-Transplantation, CHU d'Amiens, Amiens, France; Service de Néphrologie-Hémodialyse-Transplantation Rénale, CHU de Poitiers, Poitiers, France; Service de Néphrologie, Dialyse et Transplantation, CHU Limoges, Limoges, France; Service de Néphrologie, CHU Clermont-Ferrand, Clermont-Ferrand, France; Service de Néphrologie, CH Jacques Lacarin, Vichy, France; Aix Marseille Université, Assistance Publique Hôpitaux de Marseille, Hôpital Conception, Centre de Néphrologie et Transplantation Rénale, Marseille, France; Service de Néphrologie, Hôpital Foch, Suresnes, France; Institut de Transplantation Urologie Néphrologie (ITUN), Service de Néphrologie et Immunologie Clinique, CHU Nantes, Nantes, France; Assistance Publique des Hôpitaux de Paris, Service de Néphrologie et Transplantation, Hôpital Universitaire Necker-Enfants Malades, Université de Paris, Paris, France; Centre Universitaire des Maladies Rénales, Centre Hospitalier Universitaire de Caen, Caen, France; Service de Néphrologie, Dialyse et Transplantation, CHU Nice, Nice, France; Service de Néphrologie, Dialyse et Transplantation, CHU Nice, Nice, France; Service de Néphrologie et Transplantation, CHU Saint-Etienne, Saint-Etienne, France; Service de Néphrologie, CHU Angers, Angers, France; Institut de Transplantation Urologie Néphrologie (ITUN), Service de Néphrologie et Immunologie Clinique, CHU Nantes, Nantes, France; Département de Néphrologie et Transplantation d'Organes, CHU Toulouse, INSERM U1043, IFR–BMT, Université Paul Sabatier, Toulouse, France; Service de Néphrologie, CHU Clermont-Ferrand, Clermont-Ferrand, France; Assistance Publique des Hôpitaux de Paris, Service de Néphrologie et Transplantation, Hôpital Universitaire Necker-Enfants Malades, Université de Paris, Paris, France; Service de Néphrologie, CHU Clermont-Ferrand, Clermont-Ferrand, France

**Keywords:** focal segmental glomerulosclerosis, graft survival, kidney transplantation, prophylactic treatment, recurrent glomerular disease

## Abstract

**Background:**

Recurrence of focal segmental glomerulosclerosis (FSGS) is common after kidney transplantation and is classically associated with a significant decrease in graft survival. A major risk factor is a prior history of FSGS recurrence on a previous graft. This analysis reports the impact of a prophylactic treatment of FSGS recurrence in very high-risk patients who experienced a recurrence on a previous graft.

**Methods:**

We performed a retrospective multicentre observational study in 25 French transplantation centres. The inclusion criteria were patients aged more than 18 years who had undergone kidney transplant between 31 December 2004 and 31 December 2020, and who had a history of FSGS recurrence on a previous graft.

**Results:**

We identified 66 patients: 40 received prophylactic treatment (PT+), including intravenous cyclosporine and/or rituximab and/or plasmapheresis, and 26 did not receive any prophylactic treatment (PT–). The time to progression to end-stage kidney disease was similar between groups. The PT+ group was younger at FSGS diagnosis and at the time of kidney retransplantation and lost their previous graft faster. The overall recurrence rate was 72.7% (76.9% in the PT– group and 70.0% in the PT+ group, *P* = .54). At least partial remission was achieved in 87.5% of patients. The 5-year graft survival was 67.7% [95% confidence interval (CI) 53.4%–78.4%]: 65.1% (95% CI 48.7%–77.4%) in patients with FSGS recurrence vs 77.3% (95% CI 43.8%–92.3%) in patients without recurrence (*P* = .48).

**Conclusion:**

Our study suggests that prophylactic treatment should not be used routinely in patients receiving a second transplantation after recurrence of FSGS on a previous graft. The recurrence rate is high regardless of the use of prophylactic treatment. However, the 5-year graft survival remains satisfactory.

## Graphical Abstract

**Graphical Abstract:**
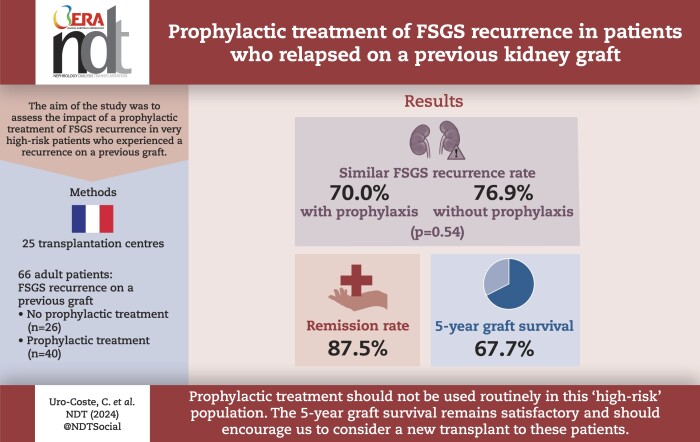

KEY LEARNING POINTS **What was known:**

- Recurrence of focal segmental glomerulosclerosis (FSGS) is common after kidney transplantation.
- The most well-known recurrence risk factor is a prior history of FSGS recurrence on a previous graft.

**This study adds:**

- This multicentre retrospective study is the first to describe the impact of prophylactic treatment in this specific, very high-risk population.
- The overall recurrence rate was 72.7% and did not differ whether or not a prophylactic treatment was used.
- The 5-year graft survival in this population was 67.7%.

**Potential impact:**

- Prophylactic treatment should not be used routinely in patients receiving a second transplantation after recurrence of FSGS on a previous graft.
- The 5-year graft survival remains satisfactory for a cohort of patients with transplant rank >1.

## INTRODUCTION

Recurrence of focal segmental glomerulosclerosis (FSGS) is common after kidney transplantation (KT), estimated to be between 10% and 60% [1–6]. In current practice, it is difficult to distinguish primary FSGS at risk of recurrence from genetic or secondary FSGS (viral, maladaptive, drug-induced, etc.), for which recurrence is much rarer [7]. The risk factors identified for recurrence of FSGS posttransplant are young age at diagnosis, rapid progression to end-stage kidney disease (ESKD) and a prior history of FSGS recurrence in a kidney allograft. In the latter case, the risk of recurrence is reported to be >80% [4, 8, 9].

The pathophysiology of primary FSGS remains poorly understood. The existence of a circulating factor that increases the permeability of glomerular capillaries, leading to disorganization of the podocyte cytoskeleton responsible for proteinuria, is now generally accepted [10–13]. Several candidates have been proposed [14, 15]. Treatment of posttransplant recurrence relies on plasmapheresis (PP) to eliminate this glomerular permeability factor, combined with corticosteroids and high-dose calcineurin inhibitors [6, 16–18]. Rituximab (RTX) is sometimes used as a first-line treatment in combination with the aforementioned therapies [18–23], as a second-line therapy to space out or interrupt PP [21], or as a salvage treatment after failure of standard therapy [24, 25].

Despite the various therapies available, FSGS recurrence has a significant negative impact on graft outcome, with 5-year graft survival ranging from 52% to 62% [2, 4, 6, 26]. Thus, the risk of graft loss is multiplied by five compared with nonrecurrent patients [6].

As a result, many patients deemed at high risk of posttransplant FSGS recurrence receive prophylactic treatment, although the data in the literature concerning its efficacy are discordant. Most of these studies are based on case reports or retrospective studies of small numbers of cases [8, 27–37]. The therapeutic strategies most frequently used in this indication are the same as those used to treat relapse, i.e. PP, RTX and per os or intravenous (IV) ciclosporin [9, 28–38].

The aim of our retrospective multicentre study was to describe French practices in terms of prophylactic treatment in patients at highest risk of recurrence with a history of FSGS recurrence on a previous kidney transplant and to assess its efficacy.

## MATERIALS AND METHODS

### Population and general data

A retrospective multicentre observational study was performed in 25 French adult transplantation centres. The inclusion criteria were patients aged more than 18 years who had undergone KT between 31 December 2004 and 31 December 2020, and who had a history of FSGS recurrence on a previous transplant.

The following demographic, clinical and biological data were collected: age, sex, ethnic origin, history of FSGS on the native kidney, history of FSGS recurrence on previous transplant(s) and time to return to dialysis, date of KT, initial immunosuppressive treatment, FSGS recurrence, treatment of recurrence and response to treatment, biological follow-up, graft survival and patient survival.

FSGS recurrence was defined as a proteinuria >2 g/day or a urine protein-to-creatinine ratio (UCPR) >2 g/g with (i) a kidney biopsy showing FSGS lesions or (ii) in patients with no residual urine output before transplant or a proteinuria <0.5 g/g before recurrence (so that proteinuria could not be explained by native kidney or previous graft FSGS recurrence) and without abnormalities explaining the proteinuria on kidney graft biopsy when it was performed.

Histological diagnoses were based on reports from pathologists at each centre, using the histological criteria defined in the Columbia classification [38].

Partial remission was defined as proteinuria <2 g/day or g/g and reduced by 50% in comparison with the initial value, and complete remission was defined as proteinuria <0.3 g/day or g/g [39].

This French retrospective and prospective registry study called ORHYATRE was approved on 25 June 2018 by our local research ethics committee (Committee for Protection of Human Subjects ‘SUD-EST VI’; IRB 00008526) and by the consultative committee on the use of health research information (14.510). No written consent was required for this study, but a notice of a no opposition letter was sent to all patients in accordance with national legislation.

### Definition of groups

**Figure 1:**
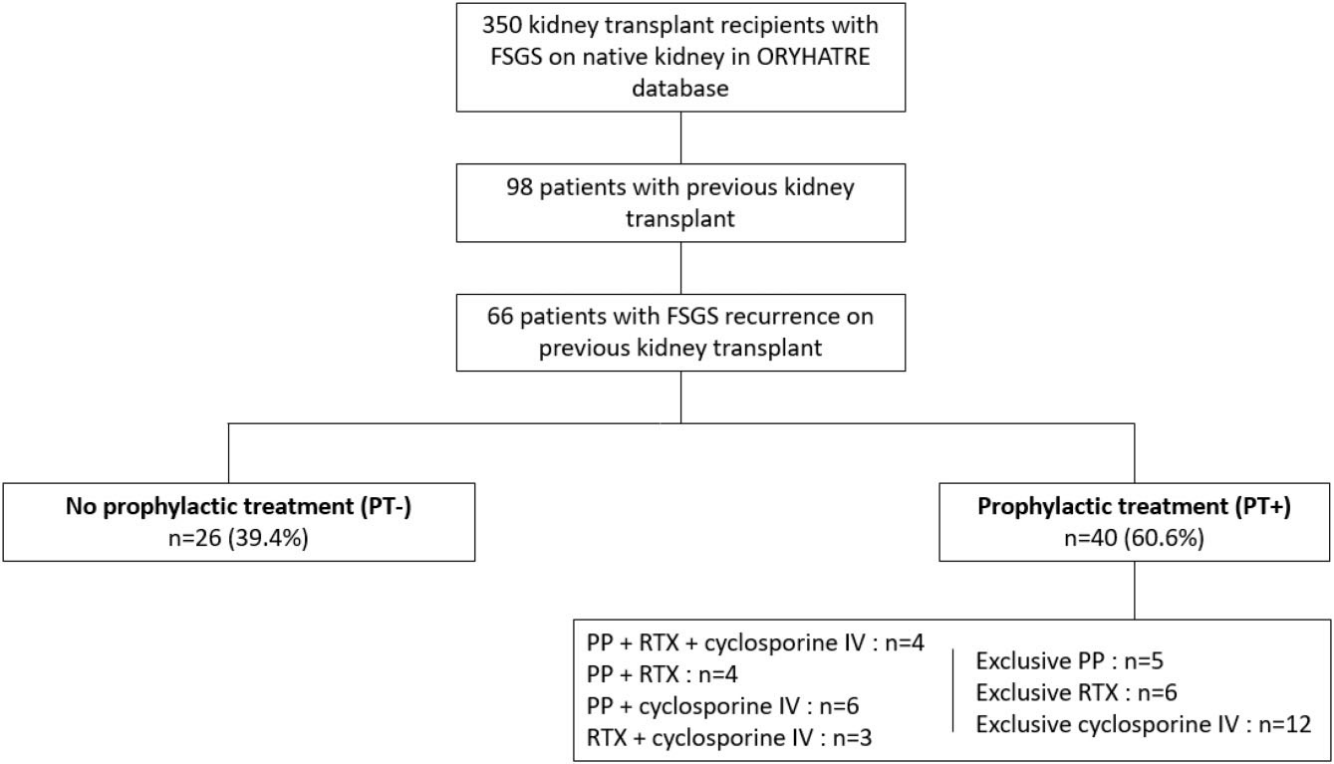
Flow chart of the study.

Our study included 66 patients who were divided into two groups: a group that received prophylactic treatment (PT+) for posttransplant FSGS recurrence, comprising 40 patients (60.6%), and a group without prophylactic treatment (PT–), including 26 patients (39.4%) (Fig. 1). The prophylactic treatment was defined by the administration of high-dose IV cyclosporine pre- and posttransplant and/or RTX in the days preceding KT or on Day 0 and/or PP in the days preceding KT or in the first days following KT, in all cases before clinical evidence of FSGS recurrence if recurrence occurred. For the latter two therapies, the indication could be the prevention of FSGS recurrence but also, as was the case for 11 patients, the desensitization of patients with preformed donor-specific antibody (DSA) or receiving an ABO-incompatible kidney allograft [40, 41]. These three different treatments could be used alone or in combination (two to three treatments). Each local team decided whether to use a preventive treatment. When treatment was carried out, the type of therapy, dosage and frequency were at the discretion of the team in charge of the patient. Details of the treatments are shown in Fig. 1.

We also studied five subgroups according to the prophylactic treatments received: (i) patients receiving RTX with or without other treatments (*n* = 17); (ii) patients receiving PP with or without other treatments (*n* = 19); (iii) patients receiving RTX and PP with or without high-dose IV cyclosporine (*n* = 8); (iv) patients receiving IV cyclosporine without other treatments (*n* = 12); and (v) patients with prophylactic treatment, excluding 12 who received only IV cyclosporine (*n* = 28).

### Statistical analysis

Statistical analyses were performed using Stata (version 15; StataCorp, College Station, TX, USA) and R (version 4.1.3; R Foundation for Statistical Computing, Vienna, Austria) softwares. All tests were two-sided with an alpha level set at 5%. Categorical variables are expressed as the number of patients and associated percentages, and continuous variables as the mean ± standard deviation or median (25th; 75th percentiles), according to their statistical distribution.

Baseline and clinical characteristics of the patients were compared between the two groups (P– and P+) using usual statistical tests: chi-squared test or Fisher's exact test for categorical variables and Student's *t*-test or Mann–Whitney test for continuous variables.

The cumulative incidence of FSGS recurrence was measured from the date of KT to the date of FSGS recurrence. Return to dialysis and death were considered competing events, and the cumulative incidence curves were compared using Gray's test.

Graft survival was a composite outcome of return to dialysis and death after KT, presented as survival free of dialysis and death. This outcome, expressed as censored data, was estimated with the Kaplan–Meier method, and the groups were compared by the log-rank statistic. Graft survival at 1 and 5 years is presented with a 95% confidence interval (95% CI).

Secondary outcomes were compared between the two groups by mixed models, considering the centre as a random effect: linear mixed models were used for continuous outcomes, and generalized linear mixed models with logit link function were used for binary outcomes.

A sensitivity analysis was performed on patients whose FSGS recurrence was confirmed by biopsy.

## RESULTS

### Patient characteristics

The characteristics of the patients are given in Table 1. The 66 patients included in our study were predominantly male (56.1%) and aged 17.6 ± 13.0 years at the time of FSGS diagnosis: 22.2 ± 15.7 years in the PT– group and 14.8 ± 10.2 years in the PT+ group (*P* = .06). The time from diagnosis to ESKD was similar between groups, with a median of 55 months (36; 84). Prior to the transplantation of interest, 16 patients (24.2%) had already received two previous grafts, and the other 50 patients (75.8%) had received only one graft. The median first-graft survival time was 37 months (17; 73) in the PT+ group and 72 months (21; 158) in the PT– group (*P* = .08). Additional data concerning previous transplants are available in Supplementary data, Table S1.

**Table 1:** Baseline and clinical characteristics of the study patients.

	**All patients (*n* = 66)**	**PT– (*n* = 26)**	**PT+ (*n* = 40)**	***P*-value**
Male sex	37 (56.1)	16 (61.5)	21 (52.5)	.47
Caucasian	56/61 (91.8)	23 (88.5)	33/35 (94.3)	.64
Age at FSGS diagnosis (years) (*n* = 57)	17.6 ± 13.0	22.2 ± 15.7	14.8 ± 10.2	.06
FSGS diagnosis at <16 years	31/57 (54.4)	10/22 (45.5)	21/35 (60.0)	.28
Time to ESKD (months) (*n* = 54)	55 (36; 84)	49 (36; 93)	57 (32; 79)	.94
FSGS immunosuppressive treatment of native kidney				
Steroids	37/54 (68.5)	13/21 (61.9)	24/33 (72.7)	.40
Steroid-sensitivity on native kidney	7/34 (20.6)	3/11 (27.3)	4/23 (17.4)	.66
Cyclosporine	22/54 (40.7)	4/21 (19.0)	18/33 (54.5)	.01
RTX	2/54 (3.7)	0/21 (0.0)	2/33 (6.1)	.52
Two previous KT	16 (24.2)	4 (15.4)	12 (30.0)	.18
Recurrence of FSGS in the first graft^a^	65 (98.5)	25 (96.2)	40 (100)	.39
Graft loss due to FSGS recurrence	46/57 (80.7)	17/22 (77.3)	29/35 (82.9)	.68
Graft survival (months) (*n* = 60)	50 (18; 100)	72 (21; 158)	37 (17; 73)	.08
Recurrence of FSGS in the second graft^b^	14/16 (87.5)	4/4 (100)	10/12 (83.3)	1.00
Graft loss due to FSGS recurrence	11/13 (84.6)	2/4 (50.0)	9/9 (100)	.08
Graft survival (months) (*n* = 14)	36 (10; 86)	46 (8; 91)	36 (14; 86)	.89
Age at KT (years)	40.2 ± 12.6	46.5 ± 13.2	36.2 ± 10.5	.002
Living donor	9/65 (13.8)	0 (0.0)	9/39 (23.1)	.009
Living related donor	8/9 (88.9)		8/9 (88.9)	NA
DSA at KT	26/60 (43.3)	11/24 (45.8)	15/36 (41.7)	.75
Induction therapy with anti-thymocyte globulin	52 (78.8)	20 (76.9)	32 (80.0)	.77
Immunosuppressive regimen				
Cyclosporine	31 (47.0)	8 (30.8)	23 (57.5)	.03
Tacrolimus	35 (53.0)	18 (69.2)	17 (42.5)	
MMF	66 (100)	26 (100)	40 (100)	NA
Steroids	66 (100)	26 (100)	40 (100)	NA
RAAS inhibitor during the first year after KT	37/50 (74.0)	17/22 (77.3)	20/28 (71.4)	.64

Data are presented as the number of patients (percentages), mean ± standard deviation or median (25th; 75th percentiles). In the first column, ‘*n*’ is the number of available data when the analyses are not performed on the whole sample (*n* = 66).

a The one patient who did not have FSGS recurrence on his first graft had a recurrence on the second.

b The two patients who did not have FSGS recurrence on the second graft had a recurrence on the first one.

MMF: mycophenolate mofetil; NA: not applicable; RAAS: renin–angiotensin–aldosterone system.

PT+ patients were younger at the time of this KT, with a mean age of 36.2 ± 10.5 years vs 46.5 ± 13.2 years in the PT– group (*P* = .002). Immunological risk and induction therapy were similar between groups; 43.3% of patients had preformed DSA and 78.8% received anti-thymocyte globulin. All living donor transplant recipients (*n* = 9) belonged to the PT+ group.

With regard to prophylaxis, the majority of patients receiving RTX (*n* = 17) received a dose of 375 mg/m^2^ with a median of 2 (1; 2) injections, and those receiving PP (*n* = 19) underwent 5.5 (1.5; 10.0) sessions. The median follow-up was 4.0 years (2.2; 7.0).

### FSGS recurrence

**Figure 2:**
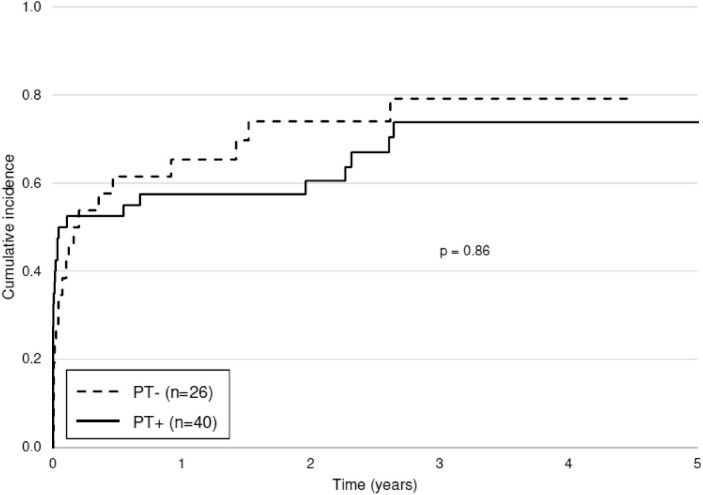
Cumulative incidence of FSGS recurrence in patients with and without prophylactic treatment. Return to dialysis and death were considered competing events.

A total of 48 patients (72.7%) experienced FSGS recurrence, without a significant difference between groups: 70.0% in the PT+ group and 76.9% in the PT– group (*P* = .54) (Table 2). The median time to recurrence was 4 days (1; 120) posttransplant in the PT+ group vs 33 days (4; 150) in the PT– group (*P* = .06). The cumulative incidence of FSGS recurrence did not differ between groups (*P* = .86), with a recurrence rate of 60.6% at 1 year posttransplant (Fig. 2). All patients who received a kidney from a living donor recurred. The recurrence rates and time to recurrence in the different patient subgroups, depending on the various prophylactic strategies, were similar to those in the PT– group (Supplementary data, Table S2).

**Table 2:** Recurrence of FSGS and clinical course of the study patients.

	**All patients** (***n* = 66)**	**PT– (*n* = 26)**	**PT+ (*n* = 40)**	***P*-value**
FSGS recurrence	48 (72.7)	20 (76.9)	28 (70.0)	.54
Time to recurrence (days) (*n* = 48)	11 (2; 150)	33 (4; 150)	4 (1; 120)	.06
Proteinuria at recurrence (g/day or UCPR) (*n* = 48)	3.0 (2.2; 6.0)	3.1 (2.2; 7.0)	3.0 (2.1; 4.9)	.62
Serum albumin at recurrence (g/dL) (*n* = 27)	32 (27; 39)	32 (28; 38)	34 (27; 39)	.34
Partial remission	42/48 (87.5)	19/20 (95.0)	23/28 (82.1)	.21
Time to partial remission (days) (*n* = 36)	20 (6; 61)	21 (11; 29)	15 (3; 132)	.46
Complete remission	26/48 (54.2)	10/20 (50.0)	16/28 (57.1)	.63
Time to complete remission (days) (*n* = 22)	36 (14; 137)	33 (12; 53)	43 (14; 183)	.58
Return to dialysis	20 (30.3)	8 (30.8)	12 (30.0)	.86
Time to return to dialysis (months) (*n* = 20)	36 (21; 50)	40 (35; 54)	32 (14; 50)	.25
For FSGS recurrence	13/20 (65.0)	5/8 (62.5)	8/12 (66.7)	.85
Death	5 (7.6)	3 (11.5)	2 (5.0)	.36

Data are presented as the number of patients (percentages) or median (25th; 75th percentiles). In the first column, ‘*n*’ is the number of available data when the analyses are not performed on the whole sample (*n* = 66).

FSGS recurrence was confirmed by kidney biopsy showing FSGS lesions in 29 of the 48 recurrence cases. The most frequent histological anomaly was podocyte alteration (55%), followed by tips lesions (20%). A sensitivity analysis of these 29 patients revealed no significant difference between the two groups, with biopsy-proven recurrence rates of 42.3% in the PT– group and 45% in the PT+ group (*P* = .8) (Table 3). In the other 19 cases of recurrence, there was no residual urine output before transplant or a proteinuria <0.5 g/g before the recurrence. Graft biopsy was performed in 12 of these 19 patients with a median delay of 41 days (2; 85). None showed any histological abnormality that could explain the presence of proteinuria >2 g/24 h or g/g in light microscopy. Electron microscopy was not used.

**Table 3:** Biopsy-proven FSGS recurrence and clinical course of the study patients (sensitivity analysis).

	**All patients (*n* = 66)**	**PT– (*n* = 26)**	**PT+ (*n* = 40)**	***P*-value**
Biopsy-proven FSGS recurrence	29 (43.9)	11 (42.3)	18 (45.0)	.80
Time to recurrence (days) (*n* = 29)	5 (1; 130)	74 (15; 335)	2 (1; 8)	.02
Proteinuria at recurrence (g/day or UCPR) (*n* = 29)	3.0 (2.2; 4.7)	3.2 (2.0; 7.0)	3.0 (2.4; 4.4)	.91
Serum albumin at recurrence (g/dL) (*n* = 18)	31 (27; 39)	31 (27;39)	31 (27;38)	.38
Partial remission	24/29 (82.8)	10/11 (90.9)	14/18 (77.8)	.38
Time to partial remission (days) (*n* = 22)	15 (3; 35)	20 (11; 62)	11 (1; 28)	.29
Complete remission	16/29 (55.2)	6/11 (54.5)	10/18 (55.6)	.96
Time to complete remission (days) (*n* = 15)	37 (18; 137)	44 (18; 54)	35 (18; 137)	.81

Data are presented as the number of patients (percentages) or median (25th; 75th percentiles). In the first column, ‘*n*’ is the number of available data when the analyses are not performed on the whole sample (*n* = 66).

### Remission and kidney transplant survival

Among the 48 patients with FSGS recurrence, remission was observed in 42 (87.5%) patients (26 complete and 16 partial) after a median of 20 days (6; 61) of treatment (Table 2). The remission rate trended higher in the PT– group (95.0% vs 82.1% in the PT+ group, *P* = .21). Eight out of nine living donor kidney recipients achieved at least partial remission.

**Figure 3:**
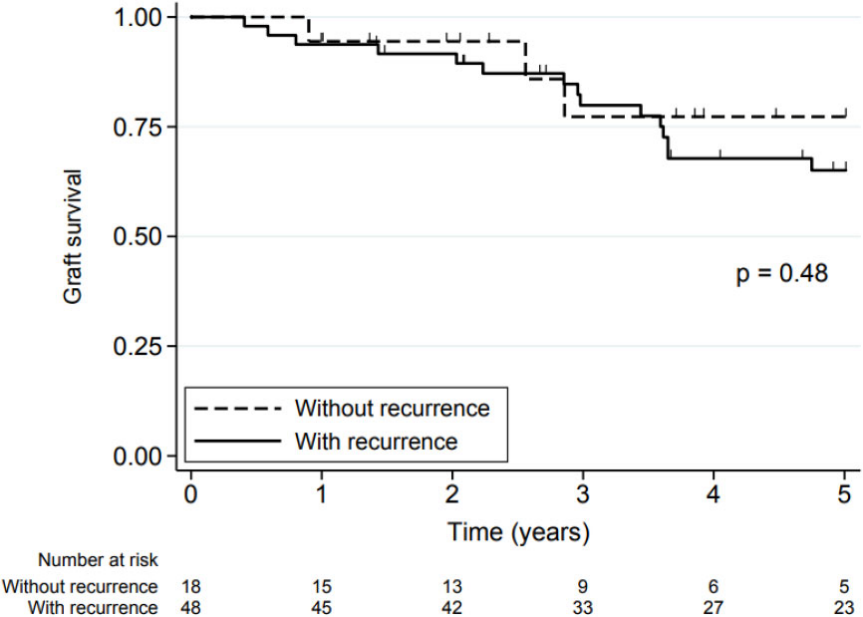
Graft survival in the study patients with and without FSGS recurrence. Graft survival was define as survival free of dialysis and death.

Graft survival (survival free of dialysis and death) was 93.9% (95% CI 84.6%–97.7%) at 1 year and 67.7% (95% CI 53.4 to 78.4%) at 5 years, and there was no significant difference in 5-year graft survival between the two groups (*P* = .83). The 5-year graft survival rate for patients with recurrence was 65.1% (95% CI 48.7%–77.4%) and for patients without recurrence was 77.3% (95% CI 43.8%–92.3%) (*P* = .48) (Fig. 3).

### Infections and patient survival rates

During the first year posttransplant, 22 patients (33.3%) had an infectious episode requiring hospitalization, 14 (35.0%) in the PT+ group and 8 (30.8%) in the PT– group (*P* = .62).

Five patients (7.6%) died during follow-up: three in the PT– group (haemorrhagic shock, COVID-19 infection and lymphoma) and two in the PT+ group (breast cancer and undetermined cause). All five patients had experienced posttransplant FSGS recurrence.

## DISCUSSION

To our knowledge, this is the first cohort used to evaluate the efficacy of prophylactic treatment in high-risk patients defined by a FSGS recurrence on a previous graft that is the most relevant factor confirming the primary nature of FSGS. This approach avoids a bias frequently encountered in this type of study, namely the selection of patients with genetic or secondary FSGS, which very rarely recurs [7]. We chose to select these patients from the 350 of our national registry because of their very high risk of recurrence (>80% according to the literature) [2, 4, 6, 26].

The use of prophylactic treatment was relatively widespread in our cohort. Sixty-one percent of patients received at least one of the three treatments (PP, RTX or IV cyclosporine), even if the indication for treatment was, in 11 cases, human leukocyte antigen desensitization or ABO-incompatible transplantation [40, 41]. This is a relatively large number, given the highly contradictory data in the literature. Owing to the absence of randomized studies and the lack of cohorts with homogeneous management, we observed a wide variety of practices in terms of the type of treatment (PP, RTX, cyclosporine), administration regimen and dosages used (Fig. 1). This is accentuated by the large number of transplant centres involved in the study and the small number of patients per centre.

Several prophylactic treatments have been evaluated in the literature [8, 27–37]. RTX is the most documented therapy. Fornoni *et al.* reported on a cohort of 41 adult kidney transplant recipients at risk of recurrence (because of their young age and rapid progression to ESKD), 27 of whom had received RTX [32]. They observed a reduction in the recurrence rate, from 64% in the group without prophylaxis to 26% for patients who received RTX, *P* < .05. Similarly, Audard *et al.* presented a series of four patients at high risk of relapse because of FSGS recurrence in the first transplant who received an infusion of RTX on the day of transplantation and in whom no recurrence was observed [27]. In contrast, Auñón *et al.* concluded that RTX administered on the day of transplantation and at 14 days posttransplant was ineffective in 12 patients from a cohort of 34 patients with primary FSGS (recurrence rate 50% in the group with RTX vs 40.9% in the group without RTX, *P* = .61) [28]. To our knowledge, only one prospective study has been carried out. It included 66 patients (including 24 patients with transplant rank higher than one), 37 of whom were selected as having a higher risk of recurrence [8]. Of these patients, 28 received prophylactic treatment with RTX combined with PP. The recurrence rate was 62% in the prophylactic group vs 51% in the group without prophylactic treatment (*P* = .21). However, the result may have been influenced by the group composition criterion, as patients who received prophylactic treatment were those with the most risk factors for recurrence (young age at diagnosis, albumin level <3 g/dL, progression to ESKD <5 years, recurrence on a previous graft).

PP, which is currently the mainstay of curative treatment for FSGS recurrence [6, 42, 43], is also being widely studied as a prophylactic treatment, as it is thought to eliminate a circulating permeability factor [43–45]. Here, again, conclusions on their efficacy in this indication are equivocal. A case series published by Ohta *et al.* reported only five recurrences in the 15 patients (33.3%) who received PP prophylaxis vs four recurrences out of six cases without prophylaxis (66.7%) (*P* = .16) [36]. However, this benefit has not been observed in other series [6, 30, 33]. Thus, Verghese *et al.* reported a similar recurrence rate in a paediatric population—that is, between 26 patients treated with PP (1–3 pretransplant then 5 posttransplant) and 31 patients without prophylaxis, with 27% and 26% recurrence, respectively (*P* = 1.00) [37]. A recent meta-analysis of 44 publications investigating the value of RTX and prophylactic PP concluded that there was no benefit from these treatments, whether RTX alone or in combination with PP, or PP alone [29].

Based on the results of our study, which only included patients who had relapsed on a previous graft, which constitutes the most well-recognized risk factor for recurrence, there would appear to be no benefit from prophylactic treatment. However, since the use of preventive treatment was at the discretion of each team, patients considered at even greater risk of recurrence were treated more frequently. Indeed, the patients in the PT+ group were younger both at the time of diagnosis of the disease and at the time of the new kidney transplant. The time to first graft loss was also significantly shorter in the PT+ group (Table 1). Furthermore, the median time to recurrence of 4 days may not have allowed the prophylactic treatment the time required for optimal efficacy but may have enabled recurrence to be managed extremely early, with ultimately similar graft survival between groups. The failure of prophylactic treatment could be attributed to its predominant use in the most severe patients, or to the presence of a higher quantity of a circulating factor explaining the very early recurrence in these patients. The recent discovery of the potential implication of anti-nephrin antibody in FSGS and its risk of recurrence post-transplant offers particularly interesting research prospects [46–48]. Determination of this antibody could help identify a population for which preventive treatment would be appropriate. In addition, it could represent an attractive treatment target, making it possible to determine whether the ‘dose of prophylaxis’ is sufficient by monitoring the evolution of the antibody titre.

We can also emphasize that in our study, recurrence of FSGS was not systematically confirmed by biopsy. The short median time between diagnosis of recurrence and biopsy for light microscopy could explain the absence of histological lesion in some patients [21]. Electron microscopy remains the gold standard for the early diagnosis of FSGS recurrence but was not widely used in current practice in France. This technique is only available in a few centres, with a turnaround time of several weeks. Nevertheless, our results are confirmed in the subgroup analysis of biopsy-proven FSGS. The total recurrence rate of 72.7%, although slightly lower than those reported in the literature [4, 9], remains high, regardless of prophylactic treatment. However, the 5-year graft survival in our cohort was 67.7% (95% CI 53.4%–78.4%), which is therefore quite close to the 5-year survival of retransplant patients in France over the 2007–21 period [74.7% (73.4–75.8) for transplantation rank = 2 and 67.5% (64.3–70.6) for transplantation rank >2] [49]. This may be explained by a high remission rate, based on the multitarget therapy combining PP, calcineurin inhibitors and high-dose corticosteroid therapy, possibly associated with RTX, as described in the prospective study of Canaud *et al.* [21, 42] which has been widely adopted by French renal transplant centres since it was published in 2010 [21].

In conclusion, our study suggests that prophylactic treatment should not be used routinely in the population of patients with a history of FSGS recurrence on a previous graft. Recent prospects in our understanding of the aetiological mechanisms of FSGS give us hope of successfully targeting a population for which preventive treatment would be of benefit. In the meantime, despite a high rate of recurrence on retransplantation, the 5-year graft survival remains satisfactory and should encourage us to consider a new transplant to these patients.

## Supplementary Material

gfae108_Supplemental_File

## Data Availability

The raw data supporting the conclusions of this article can be made available by the authors after data transfer agreement with the institution.
