# Supplementary material for: Prophylactic treatment of FSGS recurrence in patients who relapsed on a previous kidney graft
Source: Nephrol Dial Transplant. 2024 May 24;40(3):475–83. doi: 10.1093/ndt/gfae108 (PMC11879060; doi:10.1093/ndt/gfae108)
Supplement: gfae108_Supplemental_File [file gfae108_supplemental_file.docx]

**Supplemental Table 1. Characteristics of previous graft**

|  | **All patients**  **(n=66)** | **PT-**  **(n=26)** | **PT+**  **(n=40)** | **p-value** |  |
| --- | --- | --- | --- | --- | --- |
| **First graft** |  |  |  |  |  |
| Living donor | 6/55 (10.9) | 1/23 (4.3) | 5/32 (15.6) | 0.38 |  |
| Living related donor | 5/53 (9.4) | 1/23 (4.3) | 4/30 (13.3) | 0.37 |  |
| Time to FSGS recurrence (days) (n=52) | 8 [2; 10] | 20 [5; 1398] | 5 [1; 31] | 0.04 |  |
| Biopsy-proven FSGS | 38/48 (79.2) | 15/19 (78.9) | 23/29 (79.3) | 1.00 |  |
| Response to treatment |  |  |  |  |  |
| No response | 18/47 (38.3) | 7/18 (38.9) | 11/29 (37.9) |  |  |
| Partial remission | 22/47 (46.8) | 7/18 (38.9) | 15/29 (51.7) | 0.51 |  |
| Complete remission | 7/47 (14.9) | 4/18 (22.2) | 3/29 (10.3) |  |  |
| Time to return to dialysis (month) (n=65) | | 49 [18; 100] | 70 [25; 157] | 36 [17; 73] | 0.06 |
| Cause of graft loss |  |  |  |  |  |
| FSGS recurrence | 46/57 (80.7) | 17/22 (77.3) | 29/35 (82.9) | 0.68 |  |
| Rejection | 4/57 (7.0) | 1/22 (4.5) | 3/35 (8.6) |  |  |
| Allograft nephropathy | 5/57 (8.8) | 3/22 (13.6) | 2/35 (5.7) |  |  |
| Other cause | 2/57 (3.5) | 1/22 (4.5) | 1/35 (2.9) |  |  |
| **Second graft** |  |  |  |  |  |
| Living donor | 0/10 (0.0) | 0/3 (0.0) | 0/7 (0.0) | NA |  |
| Living related donor | 0/10 (0.0) | 0/3 (0.0) | 0/7 (0.0) | NA |  |
| Time to FSGS recurrence (days) (n=10) | 91 [2; 353] | 353 [61; 538] | 54 [2; 232] | 0.20 |  |
| Biopsy-proven FSGS | 10/11 (90.9) | 3/3 (100) | 7/8 (87.5) | 1.00 |  |
| Response to treatment |  |  |  |  |  |
| No response | 5/8 (62.5) | 2/2 (100) | 3/6 (50.0) |  |  |
| Partial remission | 3/8 (37.5) | 0/2 (0.0) | 3/6 (50.0) | 0.46 |  |
| Complete remission | 0/8 (0.0) | 0/2 (0.0) | 0/6 (0.0) |  |  |
| Time to return to dialysis (month) (n=13) |  |  |  |  |  |
| Cause of graft loss |  |  |  |  |  |
| FSGS recurrence | 11/13 (84.6) | 2/4 (50.0) | 9/9 (100) |  |  |
| Rejection | 1/13 (7.7) | 1/4 (25.0) | 0/9 (0.0) | 0.08 |  |
| Other cause | 1/13 (7.7) | 1/4 (25.0) | 0/9 (0.0) |  |  |
| *Data are presented as the number of patients (percentages), or median [25th; 75th percentiles]. In the first column, “n” is the number of available data when the analyses are not performed on the whole sample (n=66). NA: not applicable; PT-: no prophylactic treatment; PT+: prophylactic treatment.* | | | | |  |

**Supplemental Table 2. Recurrence of FSGS and clinical course of the study patients according to the type of prophylactic treatment.**

|  | **No preventive**  **treatment**  **(n=26)** | **With**  **RTX**  **(n=17)** | **With**  **PP**  **(n=19)** | **With**  **RTX + PP**  **(n=8)** | **Preventive treatment except exclusive cyclosporine**  **(n=28)** | **Exclusive cyclosporine**  **(n=12)** |
| --- | --- | --- | --- | --- | --- | --- |
| FSGS recurrence | 20 (76.9) | 12 (70.6) | 13 (68.4) | 6 (75.0) | 19 (67.9) | 9 (75.0) |
| Time to recurrence (days) | 33 [4; 150] | 15 [2; 515] | 1 [0; 16] | 9 [1; 846] | 8 [0; 200] | 3 [1; 6] |
| Partial remission | 19/20 (95.0) | 10/12 (83.3) | 10/13 (76.9) | 4/6 (66.7) | 16/19 (84.2) | 7/6 (77.8) |
| Complete remission | 10/20 (50.0) | 8/12 (66.7) | 6/13 (46.2) | 4/6 (66.7) | 10/19 (52.6) | 6/9 (66.7) |
| Return to dialysis | 8 (30.8) | 5 (29.4) | 6 (31.6) | 4 (50.0) | 7 (25.0) | 5 (41.7) |
| Time before dialysis (months) | 40 [35; 54] | 17 [11; 34] | 26 [11; 41] | 14 [10; 26] | 34 [11; 57] | 31 [24; 43] |
| For FSGS recurrence | 5/8 (62.5) | 3/5 (60.0) | 5/6 (83.3) | 3/4 (75.0) | 5/7 (71.4) | 3/5 (60.0) |
| *Data are presented as the number of patients (percentages), or median [25th; 75th percentiles]. FSGS: focal segmental glomerulosclerosis; PP: plasmapheresis; RTX: rituximab.* | | | | | | |
